# Contexts of occurrence of child malnutrition in the district of Villaguay, Entre Ríos, Argentina. A multivariate analysis

**DOI:** 10.1371/journal.pone.0176346

**Published:** 2017-04-25

**Authors:** María Laura Bergel Sanchís, María Florencia Cesani, Evelia Edith Oyhenart

**Affiliations:** 1 IGEVET—Instituto de Genética Veterinaria “Ing. Fernando Noel Dulout” (UNLP-CONICET LA PLATA), Facultad de Ciencias Veterinarias, UNLP, La Plata, Buenos Aires, Argentina; 2 Cátedra de Antropología Biológica IV, Facultad de Ciencias Naturales y Museo, UNLP, La Plata, Buenos Aires, Argentina; Hospital Universitario de la Princesa, SPAIN

## Abstract

The analysis of nutritional status is anthropologically important to address the complex interaction of biological, social, political, economic and cultural factors. To deepen the knowledge about contexts of occurrence of child malnutrition, we analyzed nutritional status in relation to socio-environmental conditions of residence in children between three and six years from Villaguay, Entre Ríos, Argentina. We performed a cross-sectional study of 1,435 school children of both sexes. Body weight and height were measured and prevalence of low height/age (LH/A), low weight/age (LW/A), low BMI/age (LBMI/A), overweight (Ow) and obesity (Ob) was calculated using World Health Organization reference charts. Socio-environmental information was obtained through a semi-structured survey and processed by Categorical Principal Component Analysis (CatPCA). Anthropometric data showed 1.5% LW/A, 5.2% LH/A; 0.6% LBMI/A, 20.9% Ow and 10.9% Ob. CatPCA allowed us to define four groups (G1-G4) with better (G2), middle (G1) and worst (G4) urban socio-environmental conditions and one with rural characteristics (G3). G4 presented the highest LH/A prevalence and G2 the highest Ow and Ob prevalence (P<0.05). It is concluded that since the distribution of malnutrition was not even it may dependent on the context in which children grow up. Thus, the higher the socio-economic level, the higher the incidence of overweight and obesity. Conversely, at the other end of the social scale, undernutrition and increasing weight excess remained major health problems.

## Introduction

Anthropometric indicators are widely used to evaluate growth, nutritional status and general health status of individuals and populations [1]. Anthropometric studies compare measurements of a study sample with those of reference populations [2]. Such comparisons are useful to identify cases or populations with or at risk of malnutrition and to implement public health interventions accordingly [3].

Anthropologically, nutritional status represents the complex interaction among different factors, such as biological and socio-environmental factors [4]. The former includes the specific requirements for each stage of the life cycle, whereas the latter comprises the structural elements related to food availability and access. Together, these factors define the material and symbolic context where the child grows [5].

Malnutrition is the imbalance between inadequate and excessive energy intake, and it comprises deficiency (undernutrition) and excess (overweight and obesity). Both have direct consequences on health and damage may be irreversible during growth [6].

Evidence in the literature suggests that the growth of children up to five years is similar worldwide, regardless of ethnicity [6, 7]. Therefore, changes in growth and body size may be mainly due to differences in dietary intake, socioeconomic status and living conditions [8]. In this context, undernutrition is both a cause and a consequence of poverty since its accumulated effects during childhood will result in low productivity, lower income and health problems during adulthood, thus creating a vicious cycle [9, 10]. Similarly, mothers of these children also present weight and height deficiencies as a result of inadequate dietary intakes during childhood, which may derive in intergenerational undernourishment [11].

On the other hand, excess weight is associated with higher socioeconomic status/welfare [12], despite this relationship may vary depending on whether it occurs in developed or developing countries. In fact, excess weight has long been considered exclusive to developed countries and mainly involved with the poorest populations [13–17]. However, a rapid increase of the incidence of excess weight has been lastly reported in developing countries, but associated with higher income populations [18]. Additionally, an increased prevalence of overweight and obesity has also been observed in lower socioeconomic groups [12, 19–22]. In this case, difficulties in accessing adequate food may predispose children living in impoverished environments to manifest such percent excess weight [23, 24], together with iron or muscle tissue deficiency [25, 26]. Finally, Dinsa et al. [27] further explored the association of socioeconomic condition and obesity, suggesting that obesity is a problem of rich people in low income countries, whereas a mixed situation would occur in middle-income countries.

Although food issues involve different sectors, children are the most vulnerable population not only in Argentina but also in the rest of Latin America [7, 28]. Various reports confirm the tendency towards an increasing prevalence of overweight and obesity, with a concomitant decrease in underweight and a persistent height deficit, which is known as nutrition transition [29–31]. This process is characteristic of developing countries and is mainly related to the shift from traditional diets based on fiber-rich/fat-poor starch products to fat-and sugar-rich diets and industrialized foodstuff. This, together with the advances of technology, define the obesogenic environment [32, 33]. In all, changes in the nutritional pattern are due to changes in food consumption, production and marketing accompanied by changes in life style, mainly in peripheral capitalist countries [31].

Beyond this trend, several authors have reported for Argentina the coexistence of child deficiency and excess conditions with marked differences among provinces, regions and socio-economic conditions [7, 34–38]. Therefore, in order to deepen the knowledge of the contexts of occurrence of child malnutrition, we analyzed the relation between nutritional status and socio-environmental conditions of residence in school-age children between three and six years from the department of Villaguay, Entre Ríos, Argentina.

## Materials and methods

### Ethics statement

The study aims and procedures were explained during meetings held at each school. Informed consent was signed by the children’s parents or guardians. Children whose parents did not sign the forms were not measured. In addition, the children themselves were consulted and only those who agreed (orally) were included in the study.

The research was conducted attending the principles proclaimed in the Universal Declaration of Human Rights (1948), ethical standards instituted by the Nüremberg Code (1947), the Declaration of Helsinki (1964) and subsequent amendments and clarifications, and national Law 25.326 and its amendments (Law 26.343/08), regulations and rules for the protection of personal data. This study was approved by the Bioethics Committee of the Latin American School of Bioethics (CELABE, for its acronym in Spanish; Resolution 020, Record 76).

### Population

**Fig 1:**
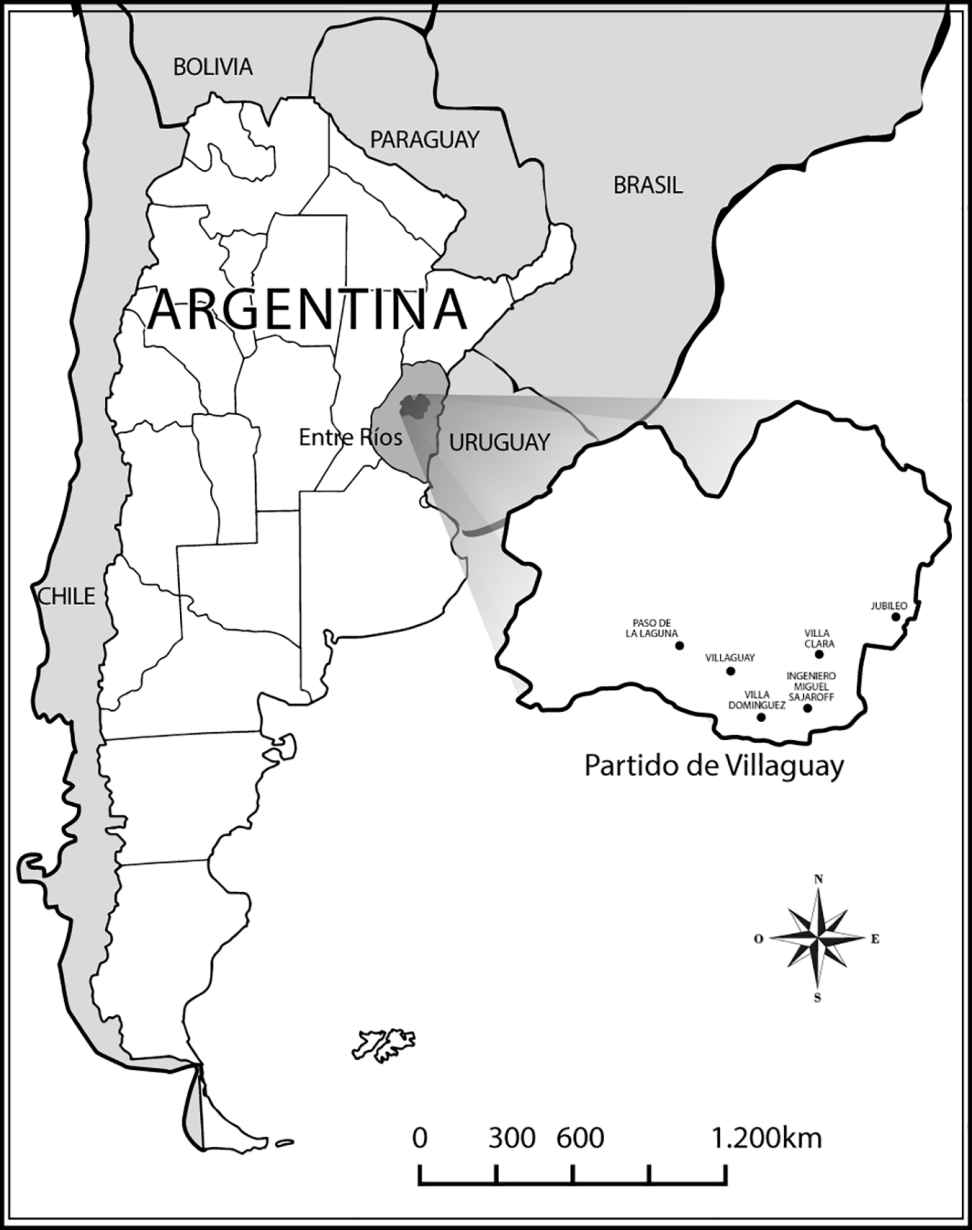
Geographic location of Villaguay department (Entre Ríos, Argentina).

The department of Villaguay is located in the center of the province of Entre Ríos, Argentina (31°51'00" S 59°01'00" W) (Fig 1). It is the fourth largest department of the province (6,753 km^2^) and the ninth with the largest population (48,965 inhabitants). Rice farming is the main economic activity, followed by livestock farming, agriculture, poultry farming, beekeeping and horticulture.

### Sample

Educational institutions were selected by convenience sampling. The total number of kindergartens and elementary public schools was recorded in six out of seven cities of the department of Villaguay (Villaguay, Villa Clara, Villa Domínguez, Jubileo, Ingeniero Sajaroff and Paso de la Laguna) in order to represent urban and rural areas.

A cross-sectional anthropometric study was performed in children aged 3.0–6.9 years. Children having a chronic disease or pathological conditions at the moment of the study were excluded. Children who did not have parental or guardian written informed consent or who refused to participate were also excluded. The final study sample included 1,435 children (722 males and 713 females), representing 47.4% of school enrolment [39] (Table 1). The study was performed during the 2010–2012 school terms.

**Table 1:** Sample composition and mean (M), median (Me) and standard deviation (SD) of the variables measured.

Age	Sample		Weight (kg)			Height (cm)			Body Mass Index		
(years)	N	%	M	Me	SD	M	Me	SD	M	Me	SD
*Male*											
3.0–3.99	82	57.8	16.33	15.87	3.86	98.18	98.05	5.17	16.86	16.56	3.26
4.0–4.99	141	45.3	18.20	17.91	3.01	104.93	105.10	5.06	16.46	16.00	1.88
5.0–5.99	264	48.9	21.16	20.18	4.49	112.24	112.40	5.76	16.66	16.21	2.31
6.0–6.99	235	53.2	22.69	21.77	4.09	116.73	116.30	5.12	16.56	16.11	2.06
**Total**	722	50.3									
*Female*											
3.0–3.99	60	42.2	15.08	14.51	3.06	96.03	96.00	5.42	16.25	15.83	2.08
4.0–4.99	170	54.7	18.19	17.23	3.88	104.43	104.00	4.91	16.61	16.08	3.09
5.0–5.99	276	51.1	19.87	19.36	3.86	110.18	110.40	6.43	16.54	15.77	5.98
6.0–6.99	207	46.8	22.38	21.62	4.47	115.82	21.62	5.45	16.58	15.98	2.40
**Total**	713	49.7									

Number (N) and percentage (%) of children surveyed and assessed, distributed by sex and age.

### Socio-environmental study

Parents or guardians completed a structured questionnaire evaluating socio-environmental characteristics and measuring housing variables with information regarding structural and physical amenities. These characteristics provided information about indoor (construction, overcrowding, main source of drinking water according to the system of water supply, sewage disposal, fuel used for cooking and heating) and outdoor housing conditions by the degree of coverage and access to public services (pavement, electricity and waste collection). To complement the information on family socioeconomic level, we asked about lodging or housing tenure, level of education and parental employment, health insurance coverage, and supplementary income, including access to national or local programs from governmental agencies, non-governmental organizations or other entities to benefit poor families by supplementing their food budget (nutritional support) and/or by providing cash relief to the heads of households (monetary support). Animal husbandry and orchard were also considered. Other aspects related to family comfort were also taken into account, such as car ownership, internet access, computer and air conditioning [40].

### Anthropometric study

Anthropometric measurements were performed by a single technician (MLBS) according to standard protocols [2]. The following variables were recorded: age, obtained from identification cards or school records; body weight (kg), measured on a digital scale (Tanita UM-061, 100 g accuracy) with children lightly clothed (to correct for this clothing, the weight of clothes was subtracted); and height (cm) measured with a portable vertical anthropometer (SECA, 1 mm accuracy).

Intra-observer coefficient error (range, 0–1) was calculated with intraclass correlation. Values greater than 0.75 were considered acceptable [41].

The exact age of each child was calculated as a function of their birth date. Similarly, body mass index (BMI = (W/H^2^) (kg/m^2^) was determined with weight and height data. Underweight (low weight-for-age, LW/A), stunting (low height-for-age, LH/A), low BMI-for-age (LBMI/A), overweight (Ow) and obesity (Ob) were determined using the World Health Organization (WHO) reference charts [42].

### Statistical analyses

Anthropometric variables were calculated as means, medians and standard deviations (Table 1). Categorical Principal Component Analysis (CatPCA) was used to process socio-environmental data. The technique is appropriate for the treatment of multivariate data of heterogeneous nature (numerical, nominal, ordinal and multinomial variables) and reduces the complexity of all socio-environmental observations related to each child without losing information [43]. CatPCA results were used to define groups of observations. The frequency of socio-environmental variables and nutritional indicators was also calculated. The latter were compared between sexes and ages using binary logistic regression and among groups defined by CatPCA by Chi-square test. Statistical processing was performed using SPSS 15.0 software.

## Results

After CatPCA analysis, the first two components represented 20.45% of the total variance. The Cronbach’s Alpha values were 0.83 and 0.70 for the first and the second axes, respectively, indicating that the original variables were adequately represented [44].

Table 2 summarizes eigenvectors from CatPCA. The most influential variables in the analysis were parental education, health insurance coverage, material and consumer goods like computer, internet, air conditioning and car, and some physical amenities such as sewer system, waste collection, bottled gas and electricity.

**Table 2:** CatPCA eigenvectors for the first two dimensions analyzed.

Variables	Dimension	
	1	2
Computer	0.689	-0.238
Mother´s education	0.666	-0.156
Internet	0.647	-0.291
Father´s education	0.638	-0.165
Air conditioning	0.614	-0.281
Health insurance	0.609	-0.112
Car	0.525	-0.200
Sewage system	0.488	0.356
Waste collection	0.479	0.398
Cable television	0.461	0.287
Mother´s work (formal employment)	0.423	-0.120
Electricity	0.418	0.517
Piped water system	0.394	0.568
Father´s work (formal employment)	0.383	0.121
Father´s work (autonomous)	0.381	-0.254
Pavement	0.369	-0.188
House building material	0.269	0.544
Mother´s work (autonomous)	0.266	-0.219
Flooring material	0.254	0.297
Piped gas	0.234	-0.440
Bottled gas	0.043	0.676
Mother´s work (laborer)	-0.040	-0.082
Monetary support	-0.041	0.163
Father´s work (retired/pensioned)	-0.045	-0.020
Protected well	-0.056	-0.298
Father´s work (unemployed)	-0.088	-0.005
Rain-tank storage	-0.104	-0.141
Firewood	-0.117	-0.044
Mother´s work (unemployed)	-0.118	0.182
Father´s work (laborer)	-0.120	0.133
Mother´s work (retired/pensioned)	-0.130	0.077
Mother´s work (informal worker)	-0.150	0.012
Orchard	-0.152	-0.063
Mother´s work (housewife)	-0.156	0.239
Kerosene	-0.156	-0.240
Animal husbandry	-0.167	-0.076
Nutritional support	-0.248	0.092
Critical crowding	-0.267	0.135
Septic tank	-0.338	-0.085
Father´s work (informal worker)	-0.409	0.167

**Fig 2:**
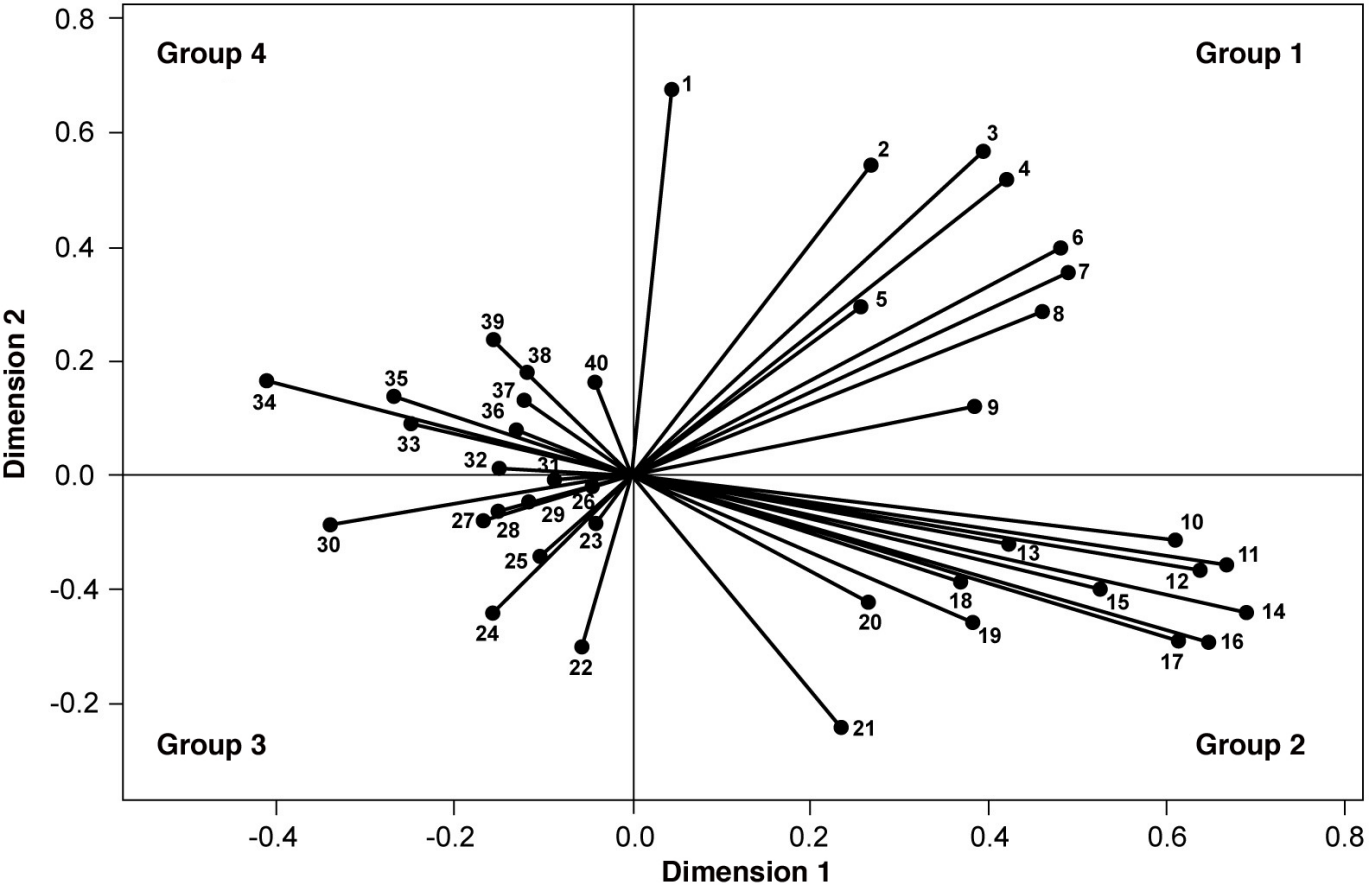
Eigenvectors corresponding to socio-environmental characteristics. Group 1: 1-Bottled gas. 2-House building material. 3-Piped water system. 4-Electricity. 5-Flooring material. 6-Waste collection. 7-Sewage system. 8-Cable television. 9-Father formal employment. Group 2: 10-Health insurance coverage. 11-Mother´s education. 12-Father´s education. 13-Mother formal employment. 14-Computer. 15-Car. 16-Internet. 17-Air conditioning. 18-Pavement. 19-Father self-employed. 20-Mother self-employed. 21-Bottled gas. Group 3: 22-Water pump. 23-Mother laborer. 24-Kerosene; 25-Water tank. 26-Animal husbandry. 27-Father retired/pensioned. 28-Orchard. 29-Firewood. 30- Septic tank. 31-Father unemployed. Group 4: 32-Mother informal employment. 33-Nutritional support. 34-Father informal employment. 35-Critical overcrowding. 36-Mother retired/pensioned. 37-Father laborer. 38-Mother unemployed. 39-Housewife. 40-Money support. Lodging status is not visible in the figure because it is a multiple nominal variable (nonlineal).

From the order established by the average values of the first two components, four groups of observations were defined (Fig 2), as follows:

Group 1 (G1, dimension-1 positive; dimension-2 positive): Families had access to public services (piped water system, electricity, sewage system, bottled gas, waste collection) and television; houses were built with fired brick masonry and flooring materials; fathers had formal employment.
Group 2 (G2, dimension-1 positive; dimension-2 negative): Families lived in neighborhoods with pavement, piped gas, greater access to material and consumer goods (computer, car, internet, air conditioning), fathers had tertiary/university education, mothers had formal employment and health insurance.
Group 3 (G3, dimension-1 negative; dimension-2 negative): Families practiced orchard agriculture and animal husbandry for personal consumption and used firewood and kerosene for heating or cooking; drinking water was obtained by protected well and rain-tank storage; excretes were removed by septic tank. Most fathers were unemployed or retired/pensioned.
Group 4 (G4, dimension-1 negative; dimension-2 positive): Both parents had informal work or fathers were laborers and retired/pensioned or mothers were unemployed or housewives. More than 45% of these families received public assistance (nutritional and/or monetary support), and 22.19% of them lived under critical crowding conditions.

The frequency of socio-environmental conditions in the total sample and by groups according to CatPCA analysis as well as their comparison by Chi^2^ is presented in Table 3.

**Table 3:** Frequency (%) of socio-environmental variables in the total sample and by groups (G1-G4). Chi-square (Chi^2^) comparison among groups.

Socio-environmental characteristics	Total	G1	G2	G3	G4	Chi^2^	p
	%	%	%	%	%		
**House building materials**	** **	** **	** **	** **	** **	** **	** **
Fired-brick masonry	79.3	90.3	85.0	46.6	83.0	191.708	0.000
Makeshift material	4.2	1.8	0.7	5.5	7.3	27.372	0.000
Low-quality prefab	3.2	2.4	2.1	6.4	3.0	9.630	0.220
Other materials	5.6	5.5	5.9	2.5	6.7	5.559	0.135
Flooring material	73.7	88.4	73.8	48.3	74.4	121.125	0.000
**Lodging or Home-tenure status**		** **	** **	** **	** **	** **	** **
House owner	61.0	63.2	62.9	52.1	62.2	9.363	0.025
Lease holder	18.4	23.2	25.2	11.0	14.8	27.695	0.000
Other (free lodging)	16.2	13.7	10.8	18.2	20.0	14.216	0.003
**Critical crowding**	12.2	4.2	2.1	14.8	22.1	100.089	0.000
**Pavement**	20.2	23.9	45.1	11.0	8.2	173.426	0.000
**Electricity**	91.6	99.7	98.6	59.7	96.3	379.269	0.000
**Waste collection**	82.7	98.7	93.4	42.4	83.6	359.778	0.000
**Drinking water (main source)**							
Piped water system	93.0	100.0	98.3	61.9	99.1	424.689	0.000
Protected well	3.2	0.0	4.9	13.1	0.2	106.069	0.000
Rain-tank storage	1.4	0.0	0.7	5.1	1.1	30.099	0.000
**Wastewater disposal**	** **	** **	** **	** **	** **		** **
Sewage system	73.8	96.1	86.0	28.0	71.8	377.241	0.000
Septic tank	17.0	2.1	8.7	37.3	23.2	156.523	0.000
**Fuel (cooking/heating)**		** **	** **	** **	** **		** **
Piped gas	9.0	0.0	32.2	14.4	0.6	280.611	0.000
Bottled gas (cylinder)	85.0	99.2	65.7	56.4	97.8	632.976	0.000
Firewood	10.4	5.3	10.1	16.1	11.6	19.877	0.000
Kerosene	1.3	0.0	0.3	6.8	0.4	64.727	0.000
**Father´s Education**	** **	** **	** **	** **	** **	** **	** **
Elementary	44.2	37.6	14.3	44.9	64.5	199.396	0.000
High School	31.3	48.9	54.9	16.5	12.7	238.957	0.000
Tertiary/University	6.9	5.8	25.9	0.4	0.4	212.196	0.000
**Mother´s Education**	** **	** **	** **	** **	** **	** **	** **
Elementary	44.2	33.4	10.5	51.7	66.5	263.345	0.000
High school	33.5	53.7	40.9	21.2	20.7	131.754	0.000
Tertiary/University	12.3	10.3	44.4	1.7	1.3	358.841	0.000
**Father´s Work**	** **	** **	** **	** **	** **	** **	** **
Formal Employed	47.7	80.5	53.5	26.7	30.5	273.429	0.000
Laborer	7.1	2.9	1.7	5.1	13.8	60.832	0.000
Self-employed worker	9.0	2.4	1.0	26.7	34.4	227.838	0.000
Informal worker	18.0	4.7	35.7	1.7	1.1	311.182	0.000
Unemployed	2.0	1.1	0.7	4.2	2.4	10.643	0.014
Retired/Pensioned	1.5	1.1	1.0	1.7	1.9	1.486	0.686
**Mother´s Work**	** **	** **	** **	** **	** **	** **	** **
Formal Employed	24.8	31.1	51.4	16.5	9.7	190.500	0.000
Laborer	0.7	0.3	0.3	1.3	0.9	3.097	0.377
Self-employed worker	3.8	0.8	1.0	7.2	6.0	29.576	0.000
Informal worker	3.8	1.6	15.7	0.8	0.4	138.403	0.000
Unemployed	8.8	7.9	2.1	3.4	15.3	53.575	0.000
Retired/Pensioned	4.2	55.8	26.9	47.5	57.2	77.362	0.000
Housewife	49.2	1.8	0.7	3.4	8.2	35.510	0.000
**Health Insurance**	48.2	71.8	85.3	24.6	21.9	444.201	0.000
**Public Assistance**							
Monetary support	23.0	24.5	13.3	14.8	30.7	42.339	0.000
Nutritional support	8.8	3.4	0.3	11.0	16.1	76.117	0.000
**Farming Practice**							
Orchard (agriculture)	6.6	3.2	4.5	14.0	6.9	30.170	0.000
Animal husbandry	7.8	2.9	6.3	13.6	9.5	26.754	0.000
**Others**			** **	** **	** **	** **	** **
Internet	24.2	30.5	74.5	4.2	1.7	601.244	0.000
Cable television	84.4	98.4	95.1	53.4	82.4	255.734	0.000
Computer	34.4	52.6	85.7	8.9	5.2	658.938	0.000
Air conditioning	20.5	22.1	68.5	4.2	0.9	568.820	0.000
Car	33.9	43.4	75.5	17.4	12.1	378.283	0.000

Results of each nutritional status indicator showed that 1.5% of children had LW/A, 5.2% LH/A, 0.6% LBMI/A, 20.9% Ow and 10.9% Ob. Prevalence of LH/A, Ow and Ob were significantly different among groups. G4 presented the highest percentages of LH/A, and G2 those of Ow and Ob (Table 4).

**Table 4:** Prevalence (%) of nutritional status indicators in the total sample and by-group. Comparison among groups (Chi^2^).

Indicators	Total	G1	G2	G3	G4	Chi^2^	p
	%	%	%	%	%		
Low weight-for-age	1.5	1.8	1.4	2.1	1.1	1.397	0.706
Low height-for-age	5.2	3.7	3.5	5.1	7.3	8.265	0.041
Low BMI-for-age	0.6	0.8	0.3	1.7	0.2	6.483	0.090
Overweight	20.9	17.9	26.2	17.4	21.8	8.994	0.029
Obesity	10.9	12.9	13.6	10.6	8.3	7.596	0.050

Age, as a factor, did not result in significant differences for all the indicators. On the contrary, boys differed from girls in Ow (boys: 23.0% *vs* girls: 18.8%) and Ob (boys: 12.7% *vs* girls: 9.1%) (Table 5).

**Table 5:** Logistic regression analysis of nutritional status by age and sex.

Indicators	Covariables	Beta	Standard error	Wald coefficient	p
Low weight-for-age	Sex	0.385	0.436	0.776	0.378
	Age	-0.038	0.208	0.034	0.852
Low height-for-age	Sex	-0.243	0.238	1.040	0.307
	Age	-0.105	0.112	0.871	0.350
Low BMI-for-age	Sex	-0.688	0.709	0.941	0.331
	Age	-0.218	0.308	0.501	0.479
Overweight	Sex	-0.257	0.131	3.892	0.049
	Age	-0.096	0.062	2.369	0.123
Obesity	Sex	-0.376	0.171	4.831	0.027
	Age	-0.044	0.081	0.303	0.581

## Discussion

The results obtained in the present study allowed us to characterize the nutritional status of the infant population from the Department of Villaguay, Entre Ríos, with reference to material and symbolic contexts where children grow. We observed a high percentage of parents with informal work, low educational level and beneficiaries of money/food aid programs. However, most families lived in their own houses made of brick with mosaic tile or concrete floors, and access to piped water system, sewage system, electricity, waste collection and cable television. In this context, more than 35% of children presented some type of malnutrition.

In our study, the prevalence of acute and chronic undernutrition was low compared with that reported for the provinces of Jujuy and Catamarca, Argentina, where poverty, unhealthy environments and poor health care were among the main underlying determinants of such condition [35]. On the other hand, according to the Argentine National Nutrition and Health Survey [28], 8.0% of children aged 6–60 months presented stunting, being Entre Ríos one of the provinces with the highest percentage. Although in our study the number of low height values recorded was lower, the prevalence of stunting in children (5.2%) shows that this form of malnutrition remains an unresolved issue.

According to UNICEF [45], higher prevalence of nutritional stunting is observed in areas with indicators associated with vulnerability, such as populations living below the poverty line and with low educational level. Consistent with the above mentioned, and despite many families received food aid or money programs, most undernourished children from Villaguay (G4) lived in overcrowded households and their parents had low educational level, informal or low-skilled works, or were unemployed.

Paradoxically, undernutrition is concomitant with excess weight, a frequent condition in various Latin American countries, including Argentina [34, 46–49]. Thus, Peña and Bacallao [50] and Monteiro et al. [17] suggest that excess weight–the other side of malnutrition–competes with global hunger. Worldwide, more than 1,600 million people have excess weight, of which 400 million become obese [6]. In this regard, the WHO recognized the global epidemic of obesity, also called globesity, in 2002. Changes in dietary habits as a result of increased refined carbohydrate and saturated fats intake are responsible for such increase [51]. Similarly, changes in physical activity patterns leading to increased sedentary lifestyles would be another cause of body weight increases [52]. Although overweight and obesity is multifactorial in origin, food intake and physical activity, known as “the big two”, would be the determinant factors [53].

In Argentina, overweight and obesity have increased markedly [28, 54, 55]. Results of a multicentric study performed by Oyhenart et al. [40] in infant populations from six Argentinian provinces showed excess weight rates (Ow and Ob) in Chubut (26%), Buenos Aires (22%), Mendoza and La Pampa (15%), Jujuy (14%) and Catamarca (11%). Thus, the excess infant weight recorded in Villaguay (31.8%) would place this population among the provinces with the higher excess weight rates, in agreement with that reported by Durán et al. [7].

Different authors have analyzed the complex relationship between excess weight and socioeconomic level [13, 15, 17]. Our results show that the socio-environmental characteristics of G2 (the group with the highest rate of children with excess weight) were more favorable than those of G4 (the group with the highest rate of malnourished children), since most parents had a high educational level, formal employment, health insurance coverage and material and consumer goods (computer, car, internet, air conditioning), all indicators of greater purchasing power. Nevertheless, high Ow and Ob prevalence in the other groups evidence the magnitude of the nutritional transition in this population.

Finally, boys had higher Ow and Ob prevalence, probably in line with that stated by Aguirre [56] concerning inter-gender relationship. This author observed that food distribution among family members may be unequal: in case of food shortage, boys are given priority in terms of quantity and quality of food, since they represent the workforce both in the present (adults) and in the future (children).

## Conclusion

In summary, at least three out of ten children from Villaguay presented either deficit or excess malnutrition, disclosing the process of nutrition transition underway. However, the distribution was not homogeneous; rather, it depended on the material and symbolic context where children grew up. Thus, the higher the family socio-economic level, the higher the incidence of overweight and obesity, whereas at the other end of the social scale, undernutrition and increasing weight excess remain serious health issues.
